# Prevention of postoperative delirium with transcranial alternating current stimulation: study protocol for a randomised controlled trial

**DOI:** 10.3389/fpsyt.2026.1907287

**Published:** 2026-09-03

**Authors:** Lin Zeng, Xiaoxue Yu, Ting Yang, Jing Liao, Yinghui Ouyang, Jun Zhou

**Affiliations:** 1 Department of Anesthesiology, Shifang People’s Hospital, Shifang, Sichuan, China; 2 Department of Anesthesiology, The Affiliated Hospital, Southwest Medical University, Luzhou, Sichuan, China

**Keywords:** elderly patients, neuromodulation, non-cardiac surgery, postoperative delirium, randomized controlled trial, transcranial alternating current stimulation

## Abstract

**Introduction:**

Postoperative delirium (POD), a common and serious neurological complication in elderly patients following non-cardiac surgery, is associated with increased risk of complications, cognitive decline, prolonged hospital stay, and higher mortality. Current prevention strategies are limited and unsatisfactory. As a non-invasive neuromodulation technique that modulates cortical oscillations and may improve age-related cognitive impairment, transcranial alternating current stimulation (tACS) emerges as a promising intervention for POD.

**Methods and analysis:**

This two-stage, randomized, double-blind, sham-controlled trial will enroll elderly patients aged ≥65 years undergoing non-cardiac surgery. In Stage 1, In Stage 1, 268 patients will be randomized to physiologically matched θ-tACS, α-tACS, γ-tACS (all 2 mA) or sham stimulation to identify the optimal frequency-plus-montage tACS protocol for POD prevention. In Stage 2, another 268 patients will receive optimal-frequency-plus-montage tACS at 1 mA, 1.5 mA, 2 mA or sham stimulation to determine the optimal intensity. Primary outcome is POD incidence (3D-CAM). Secondary outcomes include pain, cognition, sleep, anxiety and depression. Electroencephalography and blood biomarkers will be assessed. Data will be analyzed using SPSS 26.0. This study aims to develop an optimal tACS protocol for POD prevention in elderly surgical patients.

**Ethics and dissemination:**

This study was approved by the Ethics Committee of Shifang people’s hospital (202634). The results of the trial will be presented at national and international meetings relevant to the topic area and submitted to international peer-reviewed journals.

**Clinical trial registration:**

## Introduction

With the global population aging rapidly, it is projected that the number of individuals aged 65 years and older will triple by 2050, accounting for 20% of the total population (1). It is estimated that one in three surgical patients are now 65 years of age or older (2). Postoperative delirium (POD) affects up to 40% of patients over 65 and is linked to a threefold to fourfold increase in mortality, prolonged hospital stay and higher complication rates (3). However, current approaches for the prevention and treatment of POD remain limited. Pharmacological interventions, which mainly target symptom relief such as excessive agitation, lack sufficient efficacy evidence, and antipsychotics, which do not address delirium’s etiology, are of limited value (4). While non-pharmacological approaches, such as intraoperative frontal EEG-guided anesthetic/analgesic titration and perioperative optimization, are used, their efficacy remains suboptimal (5).

As a non-invasive brain stimulation technique that modulates cortical activity via oscillatory currents at specific physiological frequencies, transcranial alternating current stimulation (tACS) can alter brain oscillations’ amplitude, phase, coherence, and synchrony, thus affecting neural synchronization (6, 7). It has shown promise in treating neurological disorders such as age-related cognitive deficits and chronic pain, offering a novel avenue for POD prevention and treatment (8). To investigate the effects of different frequencies and current intensities on POD and optimize stimulation parameters, we conducted a two-stage study enrolling elderly patients aged 65 years and older who were scheduled for elective non-cardiac surgery.

The first stage aimed to explore the effects of tACS at different frequencies-plus-montage on the incidence of POD in elderly patients undergoing non-cardiac surgery. Previous research found that in 26 patients having major general anesthesia, occipital θ-wave power correlated positively with delirium severity (9). Perioperative Electroencephalography (EEG) recordings show abnormal α-band connectivity may underlie POD pathophysiology (10). Additionally, a study found that 40-Hz light stimulation halved the incidence of emergence delirium in children following sevoflurane anesthesia (11). Though using light instead of electricity, it directly validates γ-band modulation’s effect on postoperative delirium-like symptoms. Trials have demonstrated that γ-frequency (40 Hz) activity is critical for neural transmission and neural network integration (12). Furthermore, it has been proposed that abnormal γ oscillations may represent a potential neural phenotype of POD, which provides a theoretical basis for γ-band stimulation to intervene in POD (13). These findings collectively laid the foundation for the present study. Considering theta, alpha and gamma oscillations arise from distinct dominant brain regions closely related to perioperative cognitive dysfunction (12, 14, 15), we matched each frequency with its corresponding target cortex in Stage 1. This physiologically oriented pairing allows us to evaluate clinically executable integrated tACS protocols instead of testing frequency as an isolated variable.

Based on the first stage results, the second stage aimed to examine the effects of optimal-frequency-plus-montage tACS at different intensities on POD incidence in elderly non-cardiac surgery patients and identify an optimal protocol for effective POD prevention. Previous studies, which tested three low intensities of 5 Hz prefrontal tACS, found they increased θ-band and α-band energy, supporting low-intensity tACS modulation of EEG signals (15). Another study, which applied 1.0–2.0 mA γ-tACS to the primary motor cortex in young and elderly individuals, found its motor cortex modulation effect depends on intensity (16). Moreover, a study that tested 1–15 mA tACS in 11 drug-resistant epilepsy patients found intensities that directly activate deep brain nuclei like bilateral hippocampi and amygdalae, establishing the first quantitative current criteria for deep-brain tACS (17). Collectively, these findings suggest that the effects of tACS at different intensities on POD incidence may vary significantly, highlighting the necessity of investigating different current intensities.

## Methods and analysis

### Study design and setting

**Figure 1:**
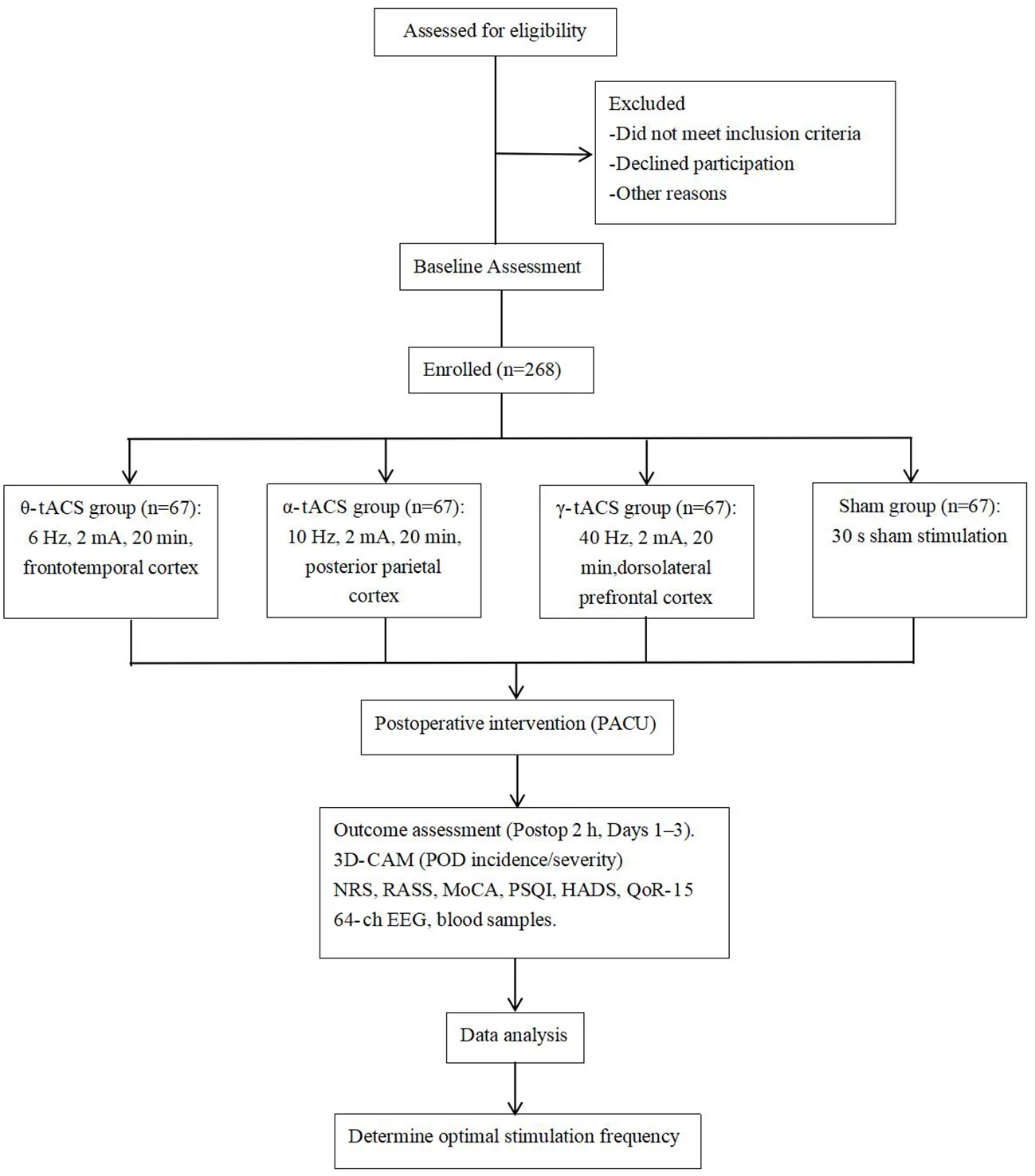
Flow chart of participant screening, grouping and procedures for the frequency-plus-montage optimization stage. Participants received theta transcranial alternating current stimulation (θ-tACS), alpha transcranial alternating current stimulation (α-tACS), gamma transcranial alternating current stimulation (γ-tACS) or sham stimulation in the post-anesthesia care unit (PACU). Baseline evaluation was conducted before intervention. The 3-minute Diagnostic Interview for Confusion Assessment Method (3D-CAM), Numerical Rating Scale (NRS), Richmond Agitation-Sedation Scale (RASS), Montreal Cognitive Assessment (MoCA), Pittsburgh Sleep Quality Index (PSQI), Hospital Anxiety and Depression Scale (HADS), 15-item Quality of Recovery (QoR15), 64-channel electroencephalography (64ch EEG) and blood tests were assessed at 2 hours and days 1–3 after surgery, followed by data analysis.

**Figure 2:**
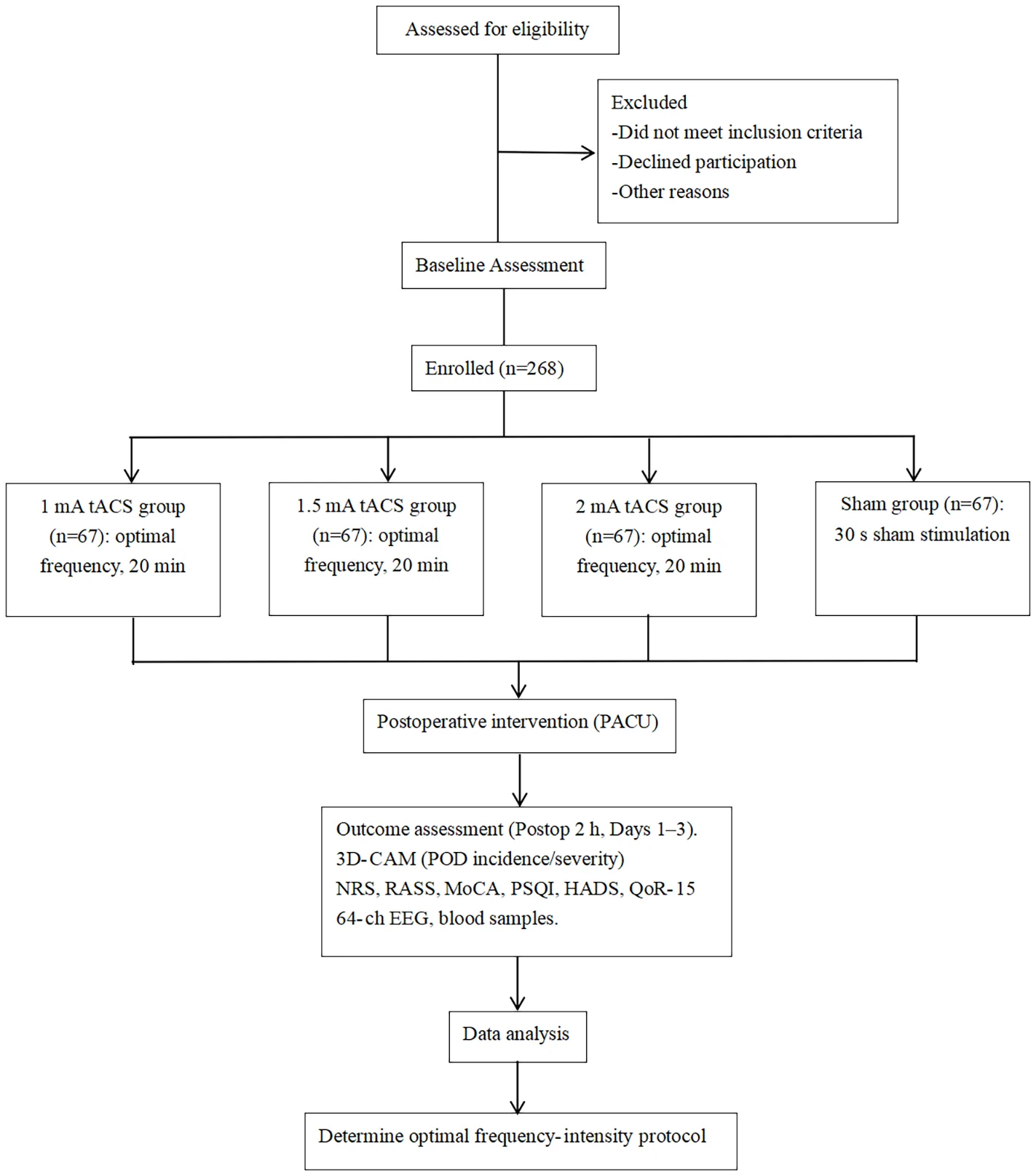
Flow chart of participant screening, grouping and procedures for the intensity optimization stage. Participants received transcranial alternating current stimulation (tACS) at 1 mA, 1.5 mA, 2 mA with the optimal frequency-plus-montage or 30-second sham stimulation in the post-anesthesia care unit (PACU). Baseline evaluation was conducted before intervention. The 3-minute Diagnostic Interview for Confusion Assessment Method (3D-CAM), Numerical Rating Scale (NRS), Richmond Agitation-Sedation Scale (RASS), Montreal Cognitive Assessment (MoCA), Pittsburgh Sleep Quality Index (PSQI), Hospital Anxiety and Depression Scale (HADS), 15-item Quality of Recovery (QoR15), 64-channel electroencephalography (64ch EEG) and blood tests were assessed at 2 hours and days 1–3 after surgery, followed by data analysis.

This is a two-stage, single-centre, prospective, randomised, double-blind, sham-controlled trial, which will be conducted from April 2026 to April 2028. Shifang People’s Hospital, which will be responsible for the recruitment, screening, intervention, and follow-up of all patients, will evaluate all outcomes. This protocol conforms to the Standard Protocol Items: Recommendations for Interventional Trials (SPIRIT) 2013 statement (18, 19). The study consists of a frequency-plus-montage optimisation phase (Stage 1) followed by an intensity optimisation phase (Stage 2). In Stage 1, we compare θ-tACS, α-tACS, γ-tACS, and sham stimulation. In Stage 2, we test three current intensities (1 mA, 1.5 mA, 2 mA) at the optimal frequency-plus-montage identified in Stage 1 and sham stimulation. The current intensity perfectly conforms to the mainstream conventional range of the 2017–2025 international guidelines (20). All participants receive standard perioperative care according to institutional protocols. The overall study design is illustrated in **Figure 1** (Stage 1) and **Figure 2** (Stage 2). The study schedule is presented in **Table 1**.

**Table 1:** Schedule of intervention and visits.

Time relative to surgery (D: day, H: hour)	D-1	H-1	Anesthesia resuscitation room	H+2	D+1	D+2	D+3
Eligibility screening	√						
Informed consent	√						
Demographic data	√						
Medical history	√						
preoperative covariate assessment	√						
tACS/sham			√				
POD (3D-CAM)		√		√	√√	√√	√√
Sedation-agitation status (RASS)		√		√	√√	√√	√√
Postoperative pain intensity (NRS)		√		√	√√	√√	√√
Sleep quality (PSQI)		√		√			√
Cognitive function (MoCA)		√		√			√
Anxiety and depression symptoms (HADS)		√		√			√
Quality of recovery (QoR-15)		√		√			√
64-channel resting-state EEG recording (5 min)		√	√		√		
Blood draw		√	√			√	

D-1: The day before surgery. H-1: One hour before surgery. H + 2: Two hours after surgery. D + 1: One day after surgery. D + 2: Two day after surgery. D + 3: Three day after surgery. tACS, Transcranial alternating current stimulation; Sham, sham stimulation; POD, Postoperative delirium; 3D-CAM, 3-Minute Diagnostic Interview for CAM-Defined Delirium; RASS, Richmond Agitation-Sedation Scale; NRS, Numerical Rating Scale; PSQI, Pittsburgh Sleep Quality Index; MoCA, Montreal Cognitive Assessment; HADS, Hospital Anxiety and Depression Scale; QoR-15, 15-item Quality of Recovery; EEG, Electroencephalography.

Stage 1: Four groups (θ-tACS, α-tACS, γ-tACS, sham), n = 67 per group, total n = 268.
Stage 2: Four groups (1 mA, 1.5 mA, 2 mA, sham), n = 67 per group, total n = 268.

### Study population

Elderly patients undergoing non-cardiac elective surgery under combined general and regional anaesthesia are eligible.

#### Formal selection rule from Stage 1 to Stage 2

The primary endpoint for stage selection is postoperative delirium (POD) incidence at 72 h postoperatively.

##### Primary selection criterion

Among the three active tACS arms (θ-tACS, α-tACS, γ-tACS) in Stage 1, the arm with the lowest observed POD incidence rate will be defined as the optimal frequency-plus-montage and carried forward to Stage 2.

##### Interim analysis timing

The interim analysis for frequency selection will be conducted only after complete enrollment, full follow-up and finalised outcome data of all 268 Stage 1 participants; no early stopping or interim look will be performed mid-recruitment.

##### Type I error control across two stages

We apply a split alpha allocation strategy to control the overall two-sided familywise type I error at α=0.05.

Stage 1 (frequency screening): α_1_ = 0.02;
Stage 2 (intensity optimisation for the single carried-forward frequency): α_2_ = 0.03;
Total α_1_+α_2_ = 0.05 to preserve the overall study-wide significance level.

##### Multiplicity adjustment within each stage

Within Stage 1, pairwise χ² comparisons between each active tACS group and sham will use the Bonferroni correction for 3 active vs sham contrasts; within Stage 2, Bonferroni correction will be applied for 3 intensity vs sham contrasts of the selected optimal frequency.

#### Inclusion criteria

1. Age ≥ 65 years, able to communicate in Chinese.
2. Expected hospital stay > 3 days.
3. American Society of Anesthesiologists (ASA) physical status II–III.
4. Elective non-cardiac surgery under combined intravenous–inhalational anaesthesia.
5. Written informed consent obtained from the patient or legal surrogate.

#### Exclusion criteria

1. History of major neurological or psychiatric disorders or Mini-Mental State Examination (MMSE) < 15.
2. Cranial or scalp injury.
3. Severe cardiovascular, hepatic, or renal dysfunction.
4. Metallic implants including pacemaker or cochlear implant.
5. Substance abuse, alcohol dependence, or severe visual/hearing impairment.
6. Expected postoperative mechanical ventilation precluding cognitive assessment.
7. Participation in another clinical trial within 1 month preoperatively.
8. Haemoglobin < 90 g/L.
9. Refusal to provide informed consent.

#### Stopping criteria for tACS

1. Headache, dizziness or scalp pain reaching moderate severity (VAS score ≥4 out of 10), which will lead to immediate termination of stimulation.
2. Any acute neurological symptoms.
3. Significant haemodynamic instability.

#### Termination criteria

1. Patient withdrawal of consent.
2. Adverse events requiring discontinuation of study intervention.
3. Protocol violation.

### Purpose of the study

#### Primary outcome

Incidence of postoperative delirium (POD) within 72 hours postoperatively, assessed via the 3D-CAM (21).

#### Secondary outcomes

1. Postoperative pain intensity (Numerical Rating Scale, NRS).
2. Quality of recovery (15-item Quality of Recovery, QoR-15).
3. Sleep quality (Pittsburgh Sleep Quality Index, PSQI).
4. Cognitive function (Montreal Cognitive Assessment, MoCA).
5. Anxiety and depression symptoms (Hospital Anxiety and Depression Scale, HADS).
6. Sedation-agitation status (Richmond Agitation-Sedation Scale, RASS).
7. Electroencephalographic changes (64-channel Electroencephalography, EEG).
8. Perioperative blood biomarkers (Visinin-like Protein-1, VILIP-1; C-C Motif Chemokine Ligand 2, CCL2; Triggering Receptor Expressed on Myeloid Cells-2, TREM-2; Interleukin-6, IL-6; Tumor Necrosis Factor-α, TNF-α).

### Randomisation and blinding

Participants are randomised using a computer-generated random number table in a 1:1:1:1 ratio. Allocation is concealed in sealed, sequentially numbered envelopes. Patients, outcome assessors, and data analysts are blinded to group assignment. Only the operator delivering tACS is unblinded. The sham stimulation uses the same electrode arrangement and current ramp protocols as all active tACS groups, lasting only 30 seconds to produce identical initial scalp tingling for participant masking. Since all tested intensities are confined to 1–2 mA with mild, comparable cutaneous sensations, the risk of broken blinding caused by distinct phosphenes or pain is greatly reduced. After intervention, participants and assessors will guess group allocation to test blinding integrity.

### Description of the study intervention

#### Stage 1

1. *θ-tACS group*: 6 Hz, 2 mA, 20 min, bilateral fronto-temporal.
2. *α-tACS group*: 10 Hz, 2 mA, 20 min, bilateral parieto-occipital.
3. *γ-tACS group*: 40 Hz, 2 mA, 20 min, bilateral dorsolateral prefrontal cortex.
4. *Sham group*: 30 s of stimulation with otherwise identical parameters.

#### Stage 2

1. *1 mA group*: Optimal frequency-plus-montage, 1 mA, 20 min.
2. *1.5 mA group*: Optimal frequency-plus-montage, 2 mA, 20 min.
3. *2 mA group*: Optimal frequency-plus-montage, 4 mA, 20 min.
4. *Sham group*: 30 s of stimulation.

Stimulation is delivered once postoperatively in the PACU after emergence from anaesthesia. Scalp is cleaned; saline-soaked sponge electrodes are secured with an elastic cap. Vital signs and adverse symptoms are monitored continuously.

### Study procedure and data collection

#### Participant screening and enrolment

Screening occurs 1 day preoperatively. Eligible patients provide written informed consent and complete baseline questionnaires.

#### Preoperative assessments

1. Demographics, medical history, and preoperative covariate assessment with validated chronic pain and trait anxiety questionnaires, including the Pain Profile Inventory (PPI), State-Trait Anxiety Inventory (STAI), Pain Catastrophizing Scale (PCS), Brief Resilience Inventory (BRI), Pain Sensitivity Questionnaire (PSQ), and Douleur Neuropathique en 4 Questions (DN4), for statistical adjustment.
2. 64-channel resting EEG (5 min).
3. Preoperative blood sampling.
4. Preoperative scales: RASS, 3D-CAM, NRS, HADS, QoR-15, PSQI, MoCA.

#### Anaesthesia, surgery and postoperative care

Standardised combined anaesthesia is administered. Postoperative care follows institutional protocols.

PACU: Single tACS intervention; post-stimulation 64-channel EEG and blood sampling.

#### Postoperative assessments during hospitalisation

1. Postoperative 2 h, Days 1–3 (twice daily): 3D-CAM, NRS, RASS.
2. Postoperative 2 h and Day 3: HADS, QoR-15, PSQI, MoCA.
3. Postoperative Day 2: Third blood sampling.

#### Follow-up assessments

No formal long-term follow-up beyond hospitalisation is planned for this trial.

#### Endpoint

1. Primary endpoint: POD incidence within 72 hours postoperatively.
2. Secondary endpoints: Pain, recovery quality, sleep, cognition, anxiety/depression, EEG changes, and biomarker levels.

### Statistics and data management

All data will be double-entered and stored in a password-protected electronic database. Statistical analyses will be performed using IBM SPSS 26.0, with two-sided testing adopted for all inferential comparisons. The overall familywise type I error across the two-stage trial is strictly controlled at α=0.05 via split allocation: α_1_=0.02 for Stage 1 frequency screening and α_2_=0.03 for Stage 2 intensity optimisation.

1. Sample size justification.

Baseline event rate: Previous perioperative cohort data reported a POD incidence of 22.2% in elderly non-cardiac surgical patients under general anaesthesia, which was set as the event rate of the sham control group (22).

Expected intervention effect magnitude: Existing randomised controlled trials of transcranial electrical neuromodulation (tDCS) for POD demonstrated that cortical electrical stimulation reduced delirium incidence from 19.7% to 4.9%, with an odds ratio of 4.761, representing a substantial protective effect against postoperative delirium (23). Although tACS and tDCS differ in neuromodulatory mechanisms, both low-intensity transcranial electrical interventions target perioperative cortical oscillatory dysfunction to mitigate delirium risk; we conservatively adopted a relative risk reduction of 50% as the expected intervention effect for tACS in this trial to avoid overestimating efficacy.

Statistical design parameters: Two-sided test, per-stage type I error adjusted by Bonferroni correction (3 active vs. sham contrasts within each stage), target statistical power = 80%.

Attrition adjustment: A 10% participant loss-to-follow-up margin was pre-specified to account for early discharge, protocol violation, withdrawal of consent or incomplete outcome assessment.

Calculation results: For a single pairwise comparison between one active tACS arm and the sham group, a minimum of 60 participants per group was required to meet the preset statistical power. After adding the 10% attrition correction, the required sample size per group was rounded up to 67 cases.

Stage 1 consists of 4 parallel groups (θ-tACS, α-tACS, γ-tACS, sham), with n=67 per group, total enrolment = 268 patients;

Stage 2 adopts another 4 parallel groups (1 mA, 1.5 mA, 2 mA optimal tACS, sham), with n=67 per group, total enrolment = 268 patients;

The overall trial will recruit 536 participants across 8 groups to fully satisfy the statistical power requirement for both frequency screening and intensity optimisation phases. This sample size design ensures adequate statistical power to detect a clinically meaningful reduction in POD incidence between each active stimulation protocol and sham control, and offsets potential bias caused by extrapolating effect sizes from tDCS literature to tACS intervention.

2. Primary outcome analysis (binary POD incidence within 72 h postoperatively).

The primary estimand is the average treatment effect of active tACS versus sham stimulation on the odds of developing POD, estimated via multivariable logistic regression adjusted for baseline age, ASA grade and preoperative cognitive status. The pre-specified primary contrasts are each active stimulation arm against the sham arm within each stage.

For multiplicity correction within each stage’s three active-versus-sham pairwise comparisons, Bonferroni adjustment is applied to control per-stage type I error; LSD *post-hoc* testing is abandoned to avoid unregulated familywise error inflation.

3. Secondary clinical outcomes.

Continuous secondary endpoints (NRS, MoCA, HADS, PSQI, QoR15, RASS) will be analysed with repeated-measures one-way ANOVA, followed by Bonferroni-corrected pairwise comparisons between each active group and sham. Categorical secondary data will be analysed with χ² or Fisher’s exact test with Bonferroni multiplicity adjustment for eight secondary clinical endpoints. Missing data will be handled via multiple imputation.

4. Exploratory neurophysiological and biomarker analyses.

All 64-channel resting-state EEG spectral/connectivity metrics and peripheral blood inflammatory biomarkers (IL-6, TNF-α, VILIP-1, CCL2, TREM2) are designated as exploratory outcomes without formal hypothesis testing for confirmatory efficacy inference. Group differences in continuous EEG and biomarker values will be explored using one-way ANOVA with descriptive reporting of effect sizes and unadjusted p-values, with all mechanistic interpretations treated as hypothesis-generating only.

5. Safety and adverse event analysis.

Adverse event rates will be summarised descriptively by study group, with serious adverse events tabulated for safety monitoring without formal comparative statistical testing.

### Ethics and dissemination

The study protocol was approved by the Ethics Committee of Shifang people’s hospital (202634). This research proposal has been approved by the Chinese Clinical Trial Registry (ChiCTR2600123512). Results will be submitted for publication in peer-reviewed journals. Individual participant data will be de-identified and stored for 5 years post-completion.

### Safety considerations

tACS is performed according to international safety guidelines. Adverse events including headache, dizziness, scalp irritation, or neurological symptoms are recorded. Serious adverse events are reported to the ethics committee immediately. No serious risks are anticipated given the low-intensity, non-invasive nature of tACS.

## Discussion

This study presents an innovative, two-stage trial designed to systematically optimize core tACS parameters targeting abnormal neural oscillations in postoperative delirium (POD). By screening the optimal stimulation frequency-plus-montage among theta, alpha and gamma bands before determining intensity, the approach refines the intervention to enhance therapeutic targeting This stepwise exploration fills the gap of lacking unified tACS protocols for POD prevention.

Our trial establishes a comprehensive multi-dimensional outcome assessment system. Apart from using 3D-CAM to evaluate the primary endpoint of POD incidence, we also combined clinical indicators including pain, cognition, sleep and mood, together with neurophysiological EEG recordings and peripheral blood inflammatory biomarkers. This integrated evaluation not only objectively verifies the clinical efficacy of tACS, but also helps interpret its underlying action mechanisms.

This research delivers promising preliminary translational implications. As a non-invasive, easy-to-operate and safe neuromodulation technique, tACS is suitable for perioperative care of elderly surgical patients. The optimised tACS protocol identified in this single-centre trial with only one postoperative stimulation session and no long-term follow-up can serve as a tentative procedural reference for future clinical exploration. Further multi-centre trials with extended follow-up data are necessary to validate its real-world efficacy before widespread routine clinical use. If verified in subsequent large-scale studies, this protocol may offer a novel non-pharmacological strategy to lower POD risk, mitigate postoperative complications, shorten hospitalisation duration and improve comprehensive perioperative care for elderly patients.

This study also has limitations. This trial utilises simple randomisation without stratification by age or surgery type, which may introduce minor baseline imbalance across four arms; stratified block randomisation will be adopted in our future multi-centre trials to address this shortcoming. Additionally, we administered only one tACS session in the PACU instead of repeated perioperative stimulation. Although this single-intervention strategy was adopted to balance clinical operability for anesthesiologists and elderly patients’ compliance, it represents a notable limitation. We were unable to compare the efficacy of single versus repeated stimulation for mitigating POD throughout the 72-hour postoperative period. Furthermore, the absence of extended long-term follow-up means the lasting cognitive benefits of tACS cannot be evaluated, which warrants further exploration in subsequent research. Our Stage 1 comparisons evaluate complete frequency-plus-montage intervention protocols, and we do not attempt to disentangle the separate effects of frequency and stimulation site in this trial. Future separate controlled trials with fixed montage will be needed to isolate the independent effect of single frequency parameters.

**Strengths and limitations of this study**

► Novel intervention design: This two-stage randomized controlled trial systematically optimizes tACS parameters (frequency-plus-montage followed by intensity), addressing the lack of standardized protocols for POD prevention.
► Rigorous study design: Adopts a double-blind, sham-controlled design to minimize bias; strict inclusion/exclusion criteria ensure homogeneous participants (elderly non-cardiac surgery patients).
► Comprehensive outcome assessment: Combines clinical outcomes (POD, pain, cognition), neurophysiology (EEG), and blood biomarkers (IL-6, TNF-α) to explore both efficacy and potential mechanisms.
► Clinical practicability: tACS is non-invasive, safe, and easy to operate, with good translational value for perioperative elderly patient management.
► The proposed investigation is not a multicentre study.
